# Enhancing the pectolinarigenin production in *Clerodendrum phlomidis* L. f. cell suspension cultures and machine learning-based predictive modeling

**DOI:** 10.3389/fpls.2026.1866545

**Published:** 2026-06-18

**Authors:** R. Praveen Kumar, S. Ramesh Kumar

**Affiliations:** 1 School of Bio Sciences and Technology, Vellore Institute of Technology, Vellore, Tamil Nadu, India; 2 Department of Horticulture and Food Science, VIT Schoool of Agricultural Innovations and Advanced Learning, Vellore Institute of Technology, Vellore, Tamil Nadu, India

**Keywords:** cell suspension cultures, *Clerodendrum phlomidis*, machine learning, pectolinarigenin, secondary metabolites

## Abstract

*Clerodendrum phlomidis* L. f. is known for its valuable secondary metabolites and their pharmacological importance. Pectolinarigenin (PEC), a natural flavonoid recognized for its promising and diverse biological properties, particularly its potent anticancer effects, faces a significant translational challenge due to its inherent scarcity and low abundance in natural sources. In this study, the first-ever development of cell suspension cultures in the genus Clerodendrum using *C. phlomidis* was reported. The study subsequently investigated the effect of salicylic acid (SA), a potent elicitor, at various concentrations on the PEC production. The UPLC quantification of elicited cultures showed that the PEC was produced maximum (343.94 ± 3.03 µg/g dry weight (DW) on 14^th^ day in the cultures supplemented with 20.0 mg/L salicylic acid, followed by 20.0 mg/L salicylic acid yielding 337.84 ± 7.11 µg/g DW on 7^th^ day. Among the three Machine Learning (ML) models employed (Logistic regression (LR), Random Forest (RF), Gradient Boosting Machine (GBM)), the R^2^ values of the best ML models, GBM and RF showed 0.953 and 0.991 for the settled cell volume and PEC yield in the elicited cultures, respectively. These novel findings successfully demonstrate the feasibility of establishing cell suspension cultures within the Clerodendrum genus and offer a practical and efficient strategy for the *in vitro* biosynthesis of PEC, a highly useful medicinal compound.

## 1 Introduction

The pharmacological potential of medicinal plants is driven by the biosynthesis of secondary metabolites, a structurally diverse class of specialized organic compounds that function independently of primary metabolism to mediate ecological and physiological adaptations (Alamgir, 2018; Bocso and Butnariu, 2022). Plants employ these compounds, which consist of alkaloids and terpenoids and polyphenols and flavonoids, as vital defense systems that protect them from both biotic and abiotic threats while they maintain their necessary ecological functions (Al-Khayri et al., 2023; Divekar et al., 2022; Goura et al., 2025). Biogenic specialized metabolites elicit diverse pharmacological effects, driven by their intrinsic antioxidant, antimicrobial, anti-inflammatory, and anticancer properties (Bhatti et al., 2022; Sadiq et al., 2024). The medicinal properties of plants prove their value as the initial basis for traditional medicine practices which continue to supply new drugs that pharmaceutical companies develop throughout the world (Ogbuagu et al., 2022; Halder and Jha, 2023).

The genus Clerodendrum (Family: Lamiaceae, formerly Verbenaceae) comprises around 240 species holds significant ethnobotanical importance (Satthaphorn et al., 2024). Several species, including *C. chinense* (Osbeck) Mabb., *C. paniculatum* L., *C. thomsoniae* Balf.f., *C. infortunatum* L., *C. volubile* P. Beauv, *C. phlomidis* L. f., and *C. indicum* (L.) Kuntze, have been widely recognized in traditional medicinal systems, like Siddha, Ayurveda, Unani, and folklore medicines for their considerable therapeutic efficacy (Helen et al., 2021; Okaiyeto et al., 2021; Prashith Kekuda et al., 2019; Khound and Devi, 2024). *Clerodendrum phlomidis* L. f. is a highly valued, deciduous shrub native to the Indian subcontinent and parts of Southeast Asia (Shareef and Prakash, 2023; Kumarasamy et al., 2025). The roots, leaves, and bark of this plant have been traditionally used to treat multiple diseases, which include inflammatory conditions, neuralgia, rheumatism, and digestive disorders, as the plant shows anti-inflammatory and antioxidant and antifungal and antipyretic and antidiabetic properties (Anitha and Kannan, 2006; Prakash, 2013; Shrivastava and Patel, 2007; Yadav and Gupta, 2014). The *C*. *phlomidis* alcoholic extract showed effective antimalarial activity against *Plasmodium falciparum* (Simonsen et al., 2001).

**Figure 1:**
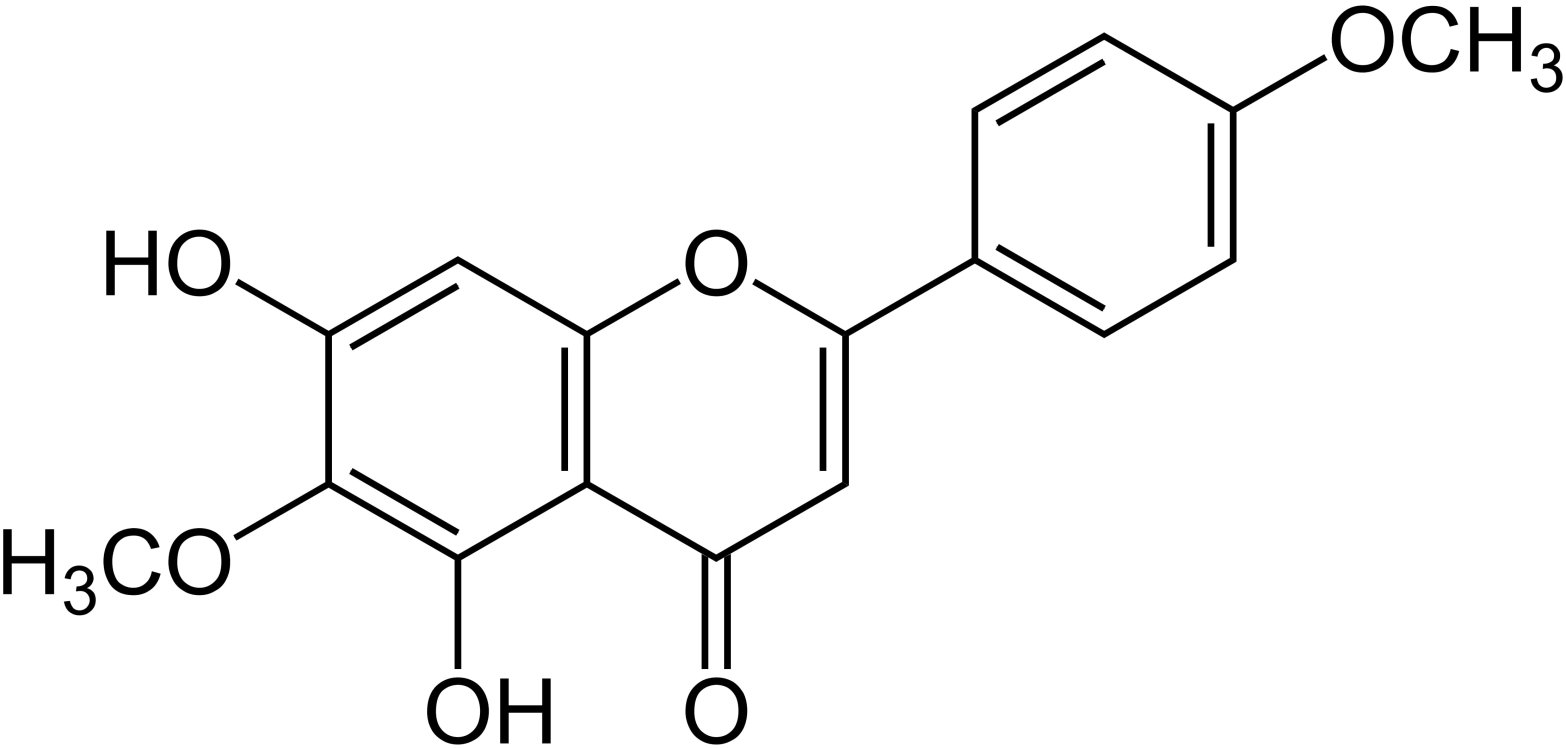
Molecular structure of pectolinarigenin (PEC).

The medicinal value of *C. phlomidis* is primarily attributed to a rich profile of secondary metabolites, with the O-methylated flavonoid Pectolinarigenin (**Figure 1**) being a key bioactive compound (Kim et al., 2018). Pectolinarigenin (PEC) (C_17_H_14_O_6_) (5,7-Dihydroxy-6,4′-dimethoxyflavone), the aglycone of pectolinarin, is a flavonoid, identified across 70 different genera and it is reported in the six species of the genus Clerodendrum (Cheriet et al., 2020; Patel and Patel, 2021; Erukainure et al., 2017). PEC possesses a wide spectrum of pharmacological properties, including potent anti-cancer, anti-oxidant, anti-inflammatory, anti-microbial, anti-diabetic, and anti-parasitic effects (Lim et al., 2008; Patel and Patel, 2021; Lee et al., 2017; Lu et al., 2014; Gan et al., 2019; Bonesi et al., 2008). Most importantly, PEC has demonstrated potent and broad-spectrum of antitumor and cytotoxic efficacy across multiple cancer models. *In vitro* and *in vivo* studies have shown promising results against challenging malignancies, including breast cancer (MCF-7), lung carcinoma (A549), melanoma (A375), osteosarcoma, gastric cancer, pancreatic cancer, and hepatocellular carcinoma (Lu et al., 2014; Bonesi et al., 2008; Lee et al., 2018; Zhou et al., 2017; Wu et al., 2018). A study by Muthu et al. (2012), reported that Pectolinarigenin isolated from *C. phlomidis* demonstrated high larvicidal efficacy against the filarial vector, *Culex quinquefasciatus*, and the dengue vector, *Aedes aegypti*.

The exhaustive harvesting for the expanding herbal trade, coupled with ongoing habitat degradation, severely compromises the conservation status of *C. phlomidis* wild populations (Aftab et al., 2025; Shukla et al., 2025). This synergistic pressure has necessitated the formal Red List designation of the species, which poses complex challenges for both conservation biology and bioprospecting initiatives (de Kok, 2021). The ongoing practice of collecting wild plants creates serious environmental problems, which directly result in major chemotype differences and unreliable access to important specialized metabolites, especially pectolinarigenin (Barbero and Maffei, 2016; Shafi et al., 2021). The current critical population decline requires immediately developed *in vitro* cultivation methods to establish better species uniformity (Babich et al., 2020; Urzì et al., 2023). The systems will function as essential components that enable successful species conservation efforts while maintaining continuous production of essential therapeutic phytochemicals at high standards.

The plant *in vitro* cell suspension culture method offers a sustainable and dependable method to produce valuable secondary metabolites through large-scale production, which does not depend on geographic or seasonal changes (Khalafalla and Mutasim, 2025; Isah et al., 2018; Wawrosch and Zotchev, 2021). The production of secondary metabolites in *in vitro* cultures reaches lower levels than what occurs in natural environments, which provide controlled conditions (Ravishankar and Venkataraman, 1993; Grzegorczyk-Karolak et al., 2016). To overcome this constraint, the strategy of elicitation is employed. Elicitors, which can be biological (biotic) or non-biological (abiotic) stress-inducing agents, when applied in specific concentrations, induce a stress response in the cultured cells, which activates their defense mechanisms and causes their secondary metabolite biosynthetic pathways to start functioning (Pusztahelyi et al., 2015; Farvardin et al., 2020; Martínez-Chávez et al., 2024). Several studies confirm this approach across various medicinal plants. The research showed that methyl jasmonate (MeJA) effectively increased flavonoid production in *Hypericum perforatum* L (Wang et al., 2015). and asiaticoside and asiatic acid synthesis in *Centella asiatica* L (Krishnan et al., 2019). Researchers employed abiotic elicitors to successfully control Echinacea purpurea (L.) Moench cichoric acid production through silver nanoparticles (Ramezannezhad et al., 2019) and used salicylic acid (SA) to boost catechin and caffeic acid and kaempferol accumulation in *Phoenix dactylifera* L (Al-Khayri and Naik, 2020) and Withaferin A and Withanolide A production in *Withania coagulans* (Stocks) Dunal (Dorrazehi et al., 2024). The combination of sodium nitroprusside with *Trichoderma harzianum* and chitosan with MeJA has been proven to enhance the production of anti-cancer alkaloids vincristine and vinblastine in *Catharanthus roseus* (L.) G.Don (Farzaei and Sayyari, 2024), and chitosan with MeJA has been shown to improve geniposide and genipin production in *Gardenia jasminoides* Ellis (Kumar and Parasurama, 2025). In *Cannabis sativa* L. adventitious root cultures, salicylic acid demonstrated a greater capacity for enhancing flavonoid accumulation compared to methyl jasmonate (Wang et al., 2024). The findings establish a strong scientific basis for the use of elicitation through signaling molecules like SA to achieve optimal pectolinarigenin production.

Machine Learning (ML), a dynamic subset of artificial intelligence, has emerged as a robust computational framework for the prediction and optimization of intricate biological systems (Saraf et al., 2024; Goshisht, 2024). In the context of *in vitro* research, leveraging ML algorithms to parse tissue culture datasets and identify optimal treatments offers a highly effective methodology (Hesami and Jones, 2021; Niazian and Niedbała, 2020; Hesami et al., 2017). Currently, sophisticated ensemble techniques specifically Random Forest (RF), Logistic Regression (LR), and Gradient Boosting Machine (GBM) are driving significant advancements across various domains of plant tissue culture (Bozkurt et al., 2024; Demirel et al., 2023; Türkoğlu et al., 2023; Hesami and Jones, 2021; Sarabandi et al., 2024; Jeevan Ram et al., 2025; Hesami et al., 2020; Kirtis et al., 2022; Jafari et al., 2022; Tütüncü, 2024; Aasim et al., 2022; Kumari et al., 2025; Ibrahim et al., 2023).

The present study aims to optimize the *in vitro* production of Pectolinarigenin using a cell suspension culture derived from *Clerodendrum phlomidis* L. f. The objectives of the study are: (i) To establish a stable and fast-growing callus cell suspension culture of *C. phlomidis*; (ii) To study the time-course effect of various concentrations of Salicylic acid (0.0 mg/L, 10.0 mg/L, 20.0 mg/L, 30.0 mg/L, and 40.0 mg/L) on the growth kinetics and Pectolinarigenin yield; and (iii) To quantify the Pectolinarigenin content in the elicited cell cultures and compare the yield to that of the field-grown plant (iv) To evaluate and select the most accurate machine learning model for predicting settled cell volume and PEC yield in elicited cultures. This research is expected to lay the groundwork for a scalable and sustainable bioprocess for Pectolinarigenin production, contributing both to pharmaceutical resource security and the *ex-situ* conservation efforts of this red-listed medicinal plant.

## 2 Materials and methods

### 2.1 Chemicals and reagents

Culture medium (MS medium (Murashige & Skoog (1962)) (with CaCl_2_), Gamborg (B5) (1968) medium, Plant Growth Regulators (PGRs) (NAA (Naphthaleneacetic acid), IAA (Indole-3-acetic acid), BAP (Benzylaminopurine), and 2,4-D (2,4-Dichlorophenoxyacetic acid)), Agar, Tween 20, and mercuric chloride (HgCl_2_) were purchased from HiMedia, Mumbai, India. Pectolinarigenin (99.79% purity) was obtained from MedChem Express, Andheri East, Mumbai, India. HPLC grade solvents (Acetonitrile, Methanol and Orthophosphoric acid) were obtained from Spectrochem Pvt. Ltd., Mumbai, India. Salicylic acid (≥99.0%) was purchased from Sigma-Aldrich, Darmstadt, Germany.

### 2.2 Plant material, explant preparation and establishment of friable calli

The plants for the current study are collected and authenticated from Siddha Medicinal Plants Garden (SMPG), Mettur dam, Salem. Callus formation was induced from leaf explants following the protocol established by Kumar and Kumar (2026). Leaf segments of *C. phlomidis* were washed with running tap water for thirty minutes, followed by Tween 20 wash for 10 minutes. Then the explants were taken into the Laminar airflow cabinet, where it was treated with 0.1% HgCl_2_ for 5 minutes and then rinsed in 70% ethanol for less than a minute. Finally, the sterilized explants were then meticulously washed with autoclaved sterile double-distilled water thrice to eliminate the residues of the sterilant.

The leaf explants were then transferred to the MS medium augmented with 3.0 mg/L NAA and 2.0 mg/L BAP. After 40 days, the developed callus was then sub-cultured in the freshly prepared MS medium with 3.0 mg/L NAA + 2.0 mg/L BAP + 1.5 mg/L 2,4-D concentrations. The cultures were incubated at 24 °C under a 16/8-hour light/dark photoperiod having a light intensity of 40 μmol m^-^² s^-^¹.

### 2.3 Establishment of suspension culture

To establish cell suspension cultures, 500 mg of five-week-old friable callus was inoculated into 60 mL of liquid Gamborg (B5) (1968) medium supplemented with 0.5 mg/L IAA, 1.0 mg/L BAP, and 1.5 mg/L 2,4-D. The flasks with cultures were agitated in a double-deck orbital shaker (Orbitek-P Pilot Double Deck Open Air Shaker) at 100rpm at 25 °C under dark conditions.

### 2.4 Growth kinetics

The cell growth of the suspension cultures was determined by calculating fresh weight (FW) in grams, dry weight (DW) in grams, and Settled Cell Volume (SCV) in percentage. All the three parameters were observed for every two days from the day of inoculation (0^th^ day) until the cells attained the decline phase. Initially, SCV was calculated by measuring the volume of settled cells in the measuring cylinder in which the culture was poured and left undisturbed for one hour. The cells were collected by Whatman No. 1 filter paper and centrifuged at 1177.25 g for 10 minutes by adding sterile double distilled water into the filtered cells to remove the media and again the cells were filtered and weighed for fresh cell weight. Then, the collected cells transferred to the oven at 50 °C for 72 hours and weighed for dry cell weight. The specific growth rate (µ, day^-1^) of the cell suspension cultures was calculated using both FW and DW data obtained across the culture period.

### 2.5 Cell morphology

The morphological characterisation of both explant-grown compact and sub-cultured friable callus cell cultures was performed using a Lawrence & Mayo LM-52–1706 upright light phase contrast microscope. Observations were systematically recorded at the time of each subculture to monitor cellular changes over time.

### 2.6 Preparation of elicitor

1mM Salicylic acid (SA) was prepared by dissolving it in ethanol. Various concentrations of SA (0.0, 10.0, 20.0, 30.0, and 40.0 mg/L) were added onto the medium of callus cell suspension culture in a sterile environment. These SA treated culture flasks were kept agitated in a shaker at 100 rpm at 25 °C under dark conditions. The effect of different concentrations of elicitor on biomass and PEC production was recorded on 0, 1, 3, 5, 7, 14, and 21 days of treatment.

### 2.7 Extraction of cell culture

The dried cells were ground into a course fine powder from which 10g was dissolved in 100ml methanol, was kept overnight in a shaker at room temperature. The mixture was then sonicated for 15 minutes and centrifuged at 817.54 g for 20 minutes and the suspension was filtered using 0.2 µm pore-size syringe filter with a polyethersulfone (PES) membrane. The extract was then used to determine the total phenolic and flavonoid content, and for the UPLC analysis for PEC quantification.

### 2.8 Determination of total phenolic and flavonoid content through spectrophotometry

The quantification of Total phenolic content (TPC) and Total flavonoid content (TFC) were calculated from the fourteenth day of inoculation. To quantify TPC, 0.2 µl of the methanolic extract was reacted with 500 µl of 10% (v/v) Folin–Ciocalteu reagent and 400 µl of 2% (w/v) sodium carbonate, followed by a 60-minute incubation in dark conditions at 25 °C to facilitate chromophore development. The resulting absorbance was measured spectrophotometrically at λ = 765 nm (Taghizadeh et al., 2019; Tahsili et al., 2014), and concentrations were extrapolated from a gallic acid standard curve. Results are expressed as milligrams of gallic acid equivalents per gram of cell dry weight (mg GAE/g DW).

To estimate TFC, 200µl of methanolic extract, 200µl of distilled water, and 120µl of 5% sodium nitrate. After incubation for 5 min in dark at 25 °C, 120µl of 10% aluminium chloride and 800µl of 1M sodium hydroxide were mixed together to read the absorbance at 415 nm (Kalemba et al., 2024). Quercetin served as the external reference standard to establish a linear calibration curve. The resulting concentrations were normalized to the sample mass and expressed as milligrams of quercetin equivalents (QE) per gram of dry weight (mg QE/g DW).

### 2.9 UPLC quantification of pectolinarigenin content

The PEC standard stock solution of mg/mL was prepared using methanol. Stock solutions of 10-100 μg/mL concentrations were prepared from the original stock solution. All the stock were stored at −4 °C. Validation of the UPLC method yielded a calibration curve regression coefficient (R^2^) of >0.991, ensuring analytical linearity with LOD and LOQ values for PEC determined to be 6.07 μg/mL and 18.40 μg/mL, respectively.

Chromatographic analysis was performed using a Waters Acquity H-class ultra-performance liquid chromatograph (UPLC) equipped with a photodiode array (PDA) detector set to scan from 190 to 800 nm. A 10.00 μL sample was injected for each analysis, and the total run time was 30.0 minutes. Chromatographic analysis was performed on a 2.5 µm BEH C18 column (4.6×150 mm) with a mobile phase consisting of 0.1% orthophosphoric acid in water (solvent A) and acetonitrile (solvent B). The gradient program was as follows: the initial ratio of A:B was 95:5 at 0 minutes, which was linearly changed to 5:95 at 30 minutes. The system was then returned to the initial conditions (95:5) at 35 minutes to re-equilibrate for the next injection. Detection of PEC was carried out at a wavelength of 284 nm. The chromatographic peak was confirmed by comparing both the retention time (RT) and the UV absorption spectrum of the sample’s peak with that of a reference standard of PEC.

### 2.10 Machine learning models

This study investigated the efficacy of three distinct machine learning (ML) algorithms, (Logistic Regression (LR), Random Forest (RF), and Gradient Boosting Machine (GBM) for modeling and predicting the SCV and PEC content in elicited cultures. The dataset comprised 42 points, with the input variables consisting of different concentrations of salicylic acid (Control, 10.0 mg/L, 20.0 mg/L, 30.0 mg/L, and 40.0 mg/L). Leave-one-out cross-validation (LOO-CV) technique was used. To enhance model robustness and generalizability, *k*-fold cross-validation was employed. This technique involved dividing the dataset into *k* subsets, enabling multiple iterations of model training and validation using varied combinations of training and testing data. The computational workflow integrated several R packages, including tidyverse for data manipulation, caret, gbm, randomForest, and ModelMetrics for predictive modeling and performance evaluation, and ggplot2 and ggpubr for high-resolution graphics. Upon completion of the training phase, the model’s performance was rigorously assessed using the dedicated testing set. Prediction accuracy was quantified using three key metrics, including the Root Mean Squared Error (RMSE), the Coefficient of Determination (R²), and the Mean Absolute Error (MAE) (Equations 1–3).

(1) $${\text{R}}^{2}=1-\;\frac{\sum_{i=1}^{n}\left(\text{yi}-\hat{y}\right)2}{\sum_{i=1}^{n}\left(\text{yi}-\hat{\overset{⌣}{y}}\right)2}$$

(2) $$\text{RMSE}=\sqrt{\sum_{i=1}^{n}{\left(yi-\hat{y}i\right)}^{2}}/n$$

(3) $$\text{MAE}=1/n\sum_{i=1}^{n}\left|yi-\hat{y}i\right|$$

Where *Yi* represents the actual value, *Yi* denotes the anticipated value, Ỹ is the mean of the actual values, as well as n is the number of data points.

### 2.11 Statistical analysis

The completely randomized design (CRD) was used to study the influence of the elicitation on the PEC production. The statistical analysis was performed on the research data using a one-way analysis of variance (ANOVA). A two-way ANOVA was performed for the elicitation studies. *Post-hoc* Duncan multiple range test (DMRT) was performed to determine specific variation between the means of groups, wherein p ≤ 0.05 is the significance level. The data were analysed using IBM SPSS Statistics (27.0.1) software, and the resulting analytical findings were based on an average of triplicates (three independent flasks) per treatment, which is presented as mean ± standard error (SE).

The performance metrics of the ML models were evaluated using RStudio software (version 4.5.1).

## 3 Results and discussion

### 3.1 Establishment of friable callus

**Figure 2:**
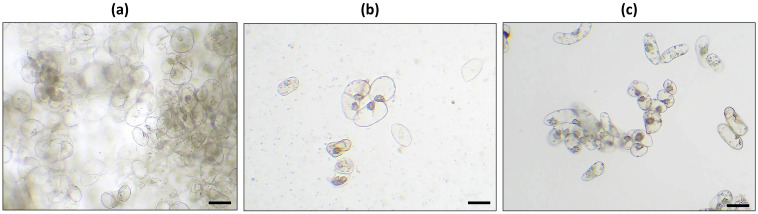
Microscopic images of **(a)** green compact callus with dense cytoplasm and **(b, c)** faded yellow friable callus during their dividing stage. Scale, 40µm.

The primary calli derived from *C. phlomidis* leaf explants were systematically sub-cultured onto MS medium supplemented with 3.0 mg/L NAA, 2.0 mg/L BAP, and 1.5 mg/L 2,4-D at 5-week intervals to induce friability. While the primary explant-derived callus exhibited a distinct green coloration characterized by tightly packed, highly aggregated cell clumps, a phenotypic transition occurred during the second sub-culture cycle. This passage successfully yielded a distinct culture of pale yellowish, friable callus characterized by a loosely arranged cellular matrix, rendering it ideal for subsequent suspension culture initiation (**Figure 2**).

### 3.2 Cell suspension cultures - growth curve analysis

**Figure 3:**
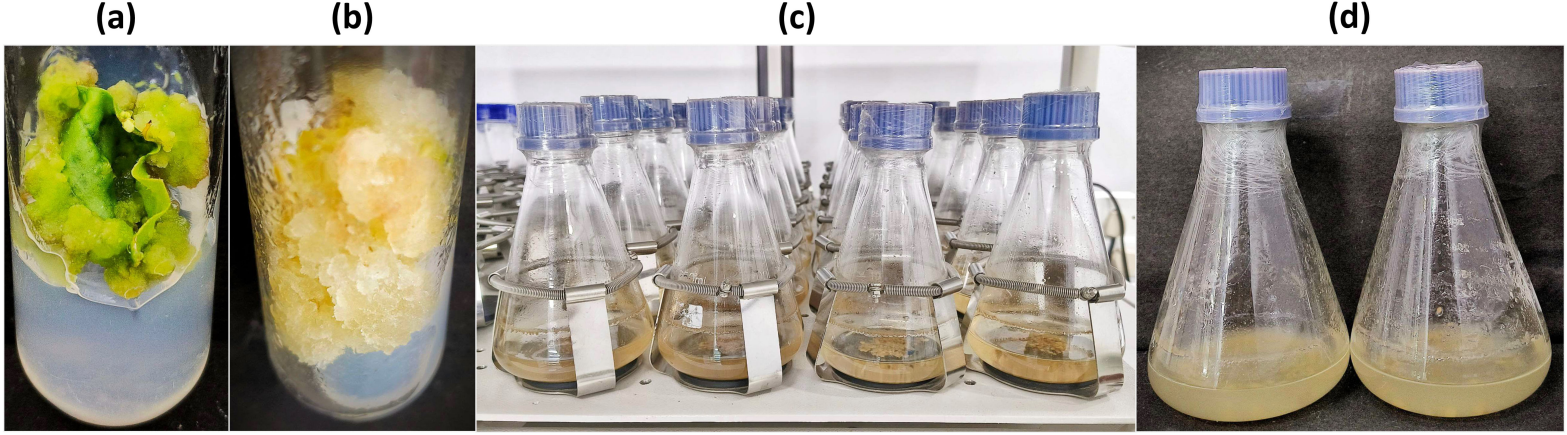
Cell suspension cultures of *Clerodendrum phlomidis*: **(a)** callus developed from leaf explants; **(b)** friable callus; **(c)** suspension cultures in an orbital shaker; **(d)** cultures after 22 days.

**Figure 4:**
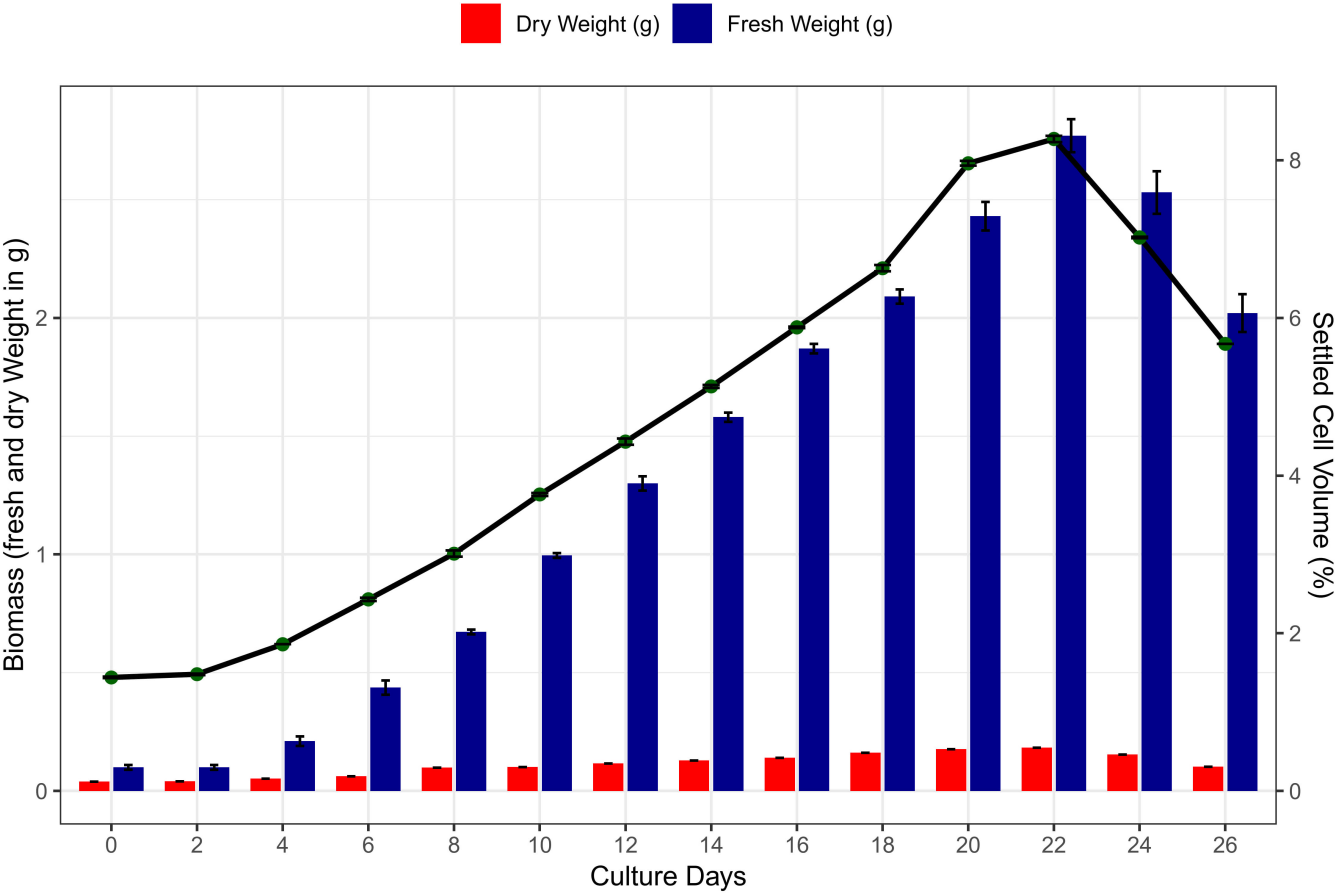
Growth curve analysis of *C. phlomidis* cell suspension cultures based on the estimation of fresh weight, dry weight, and settled cell volume (SCV).

The cell suspension cultures of *C. phlomidis* were established by transferring actively proliferating friable calli in B5 medium strengthened with IAA (0.5 mg/L), BAP (1.0 mg/L), and 2,4-D (1.5 mg/L) (**Figure 3**). The growth dynamics of the cell suspension cultures were characterized over a 26-day period by monitoring FW, DW, and SCV, revealing a typical sigmoidal growth profile with distinct lag, exponential, and decline phases. Following an initial lag phase of approximately 4 days marked by slow biomass accumulation, the cultures entered a robust exponential phase where FW, DW, and SCV increased Subsequently. The culture reached its maximum growth potential on day 22, achieving peak values of approximately 2.8 g for FW, 0.18 g for DW, and 83% for SCV. Subsequent to this stationary peak, a marked decline phase was observed after 22^nd^ day among all parameters (**Figure 4**), indicating a inhibition of growth and likely cell lysis due to nutrient depletion or metabolic byproduct accumulation. Prior to the onset of the decline phase on day 22, the culture exhibited a specific growth rate (µ) of 0.151 day^-1^ (FW) and 0.0703 day^-1^ (DW).

### 3.3 Total phenolic and flavonoid content determination

**Figure 5:**
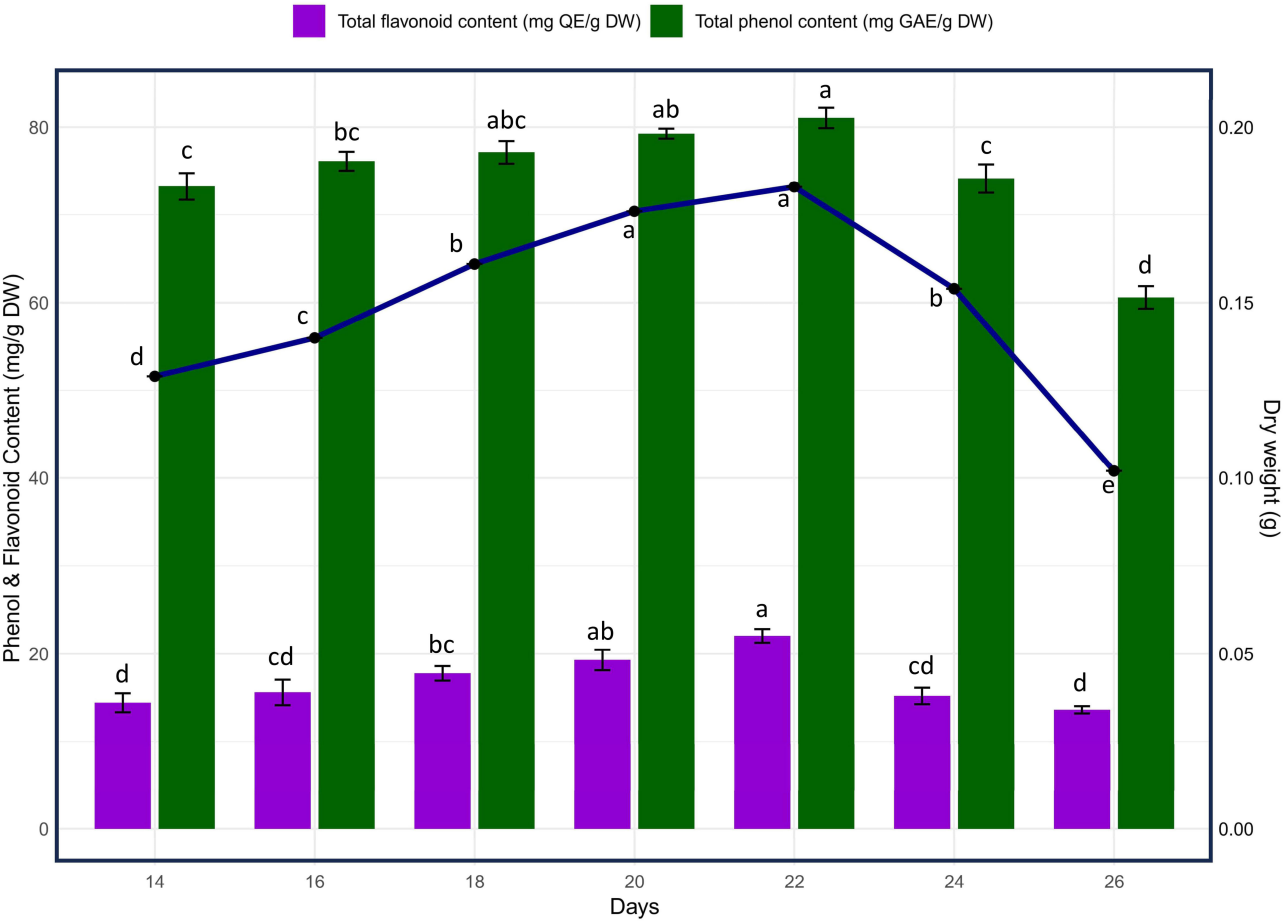
Estimation of TPC and TFC contents in suspension cultures of *C. phlomidis* developed in B5 medium augmented with 0.5 mg/L IAA, 1.0 mg/L BAP and 1.5 mg/L 2,4-D. Error bars represent Mean ± Standard Error (SE).

Plants synthesize phenolic compounds that include various flavonoids as their primary group of natural secondary metabolites (Mutha et al., 2021; Zagoskina et al., 2023; Elshafie et al., 2023). The shikimate and phenylpropanoid pathways deliver phenylpropanoids which plant phenolic components originate from phosphoenolpyruvate erythrose-4-phosphate while acetyl-Co A generates phenolic components through the acetate-malonate pathway (Kuljarusnont et al., 2024; Hajam et al., 2024). The process of flavonoid accumulation within plants establishes a crucial mechanism which safeguards plants against damage from both biotic and abiotic sources (Zhuang et al., 2023; Jan et al., 2021). The antioxidant system contains flavonoids as its main active component (Hassanpour and Doroudi, 2023). The established cell suspension culture of *C. phlomidis*, developed in B5 medium optimally supplemented with 0.5 mg/L IAA, 1.0 mg/L BAP and 1.5 mg/L 2,4-D achieved its maximum secondary metabolite production on the 22^nd^ day of culture (**Figure 5**). Specifically, this time point yielded the highest levels of TPC, quantified at 81.06 ± 1.17 mg GAE/g DW, and TFC, measured at 22.06 ± 0.78 mg QE/g DW. A direct positive correlation was observed between the accumulation of both TPC and TFC and the corresponding increase in biomass (both FW and DW) up until the 22^nd^ day, after which both metabolite yield and biomass accumulation declined, suggesting that secondary metabolite production in this system is intimately linked to the cellular growth phase, likely corresponding to the late exponential or early stationary phase.

### 3.4 Effect of salicylic acid on biomass and pectolinarigenin production

The suspension cultures developed in B5 medium augmented with 0.5 mg/L IAA, 1.0 mg/L BAP and 1.5 mg/L 2,4-D was subjected to various concentrations (10.0 mg/L, 20.0 mg/L, 30.0 mg/L, and 40.0 mg/L) of salicylic acid. The process of initiating elicitation during the lag phase leads to improved cellular priming and physiological acclimation, which results in increased metabolic flow and stress-response signaling needed to maintain secondary metabolite production from elicitors during the subsequent growth stages (Kumar and Parasurama, 2025).

**Figure 6:**
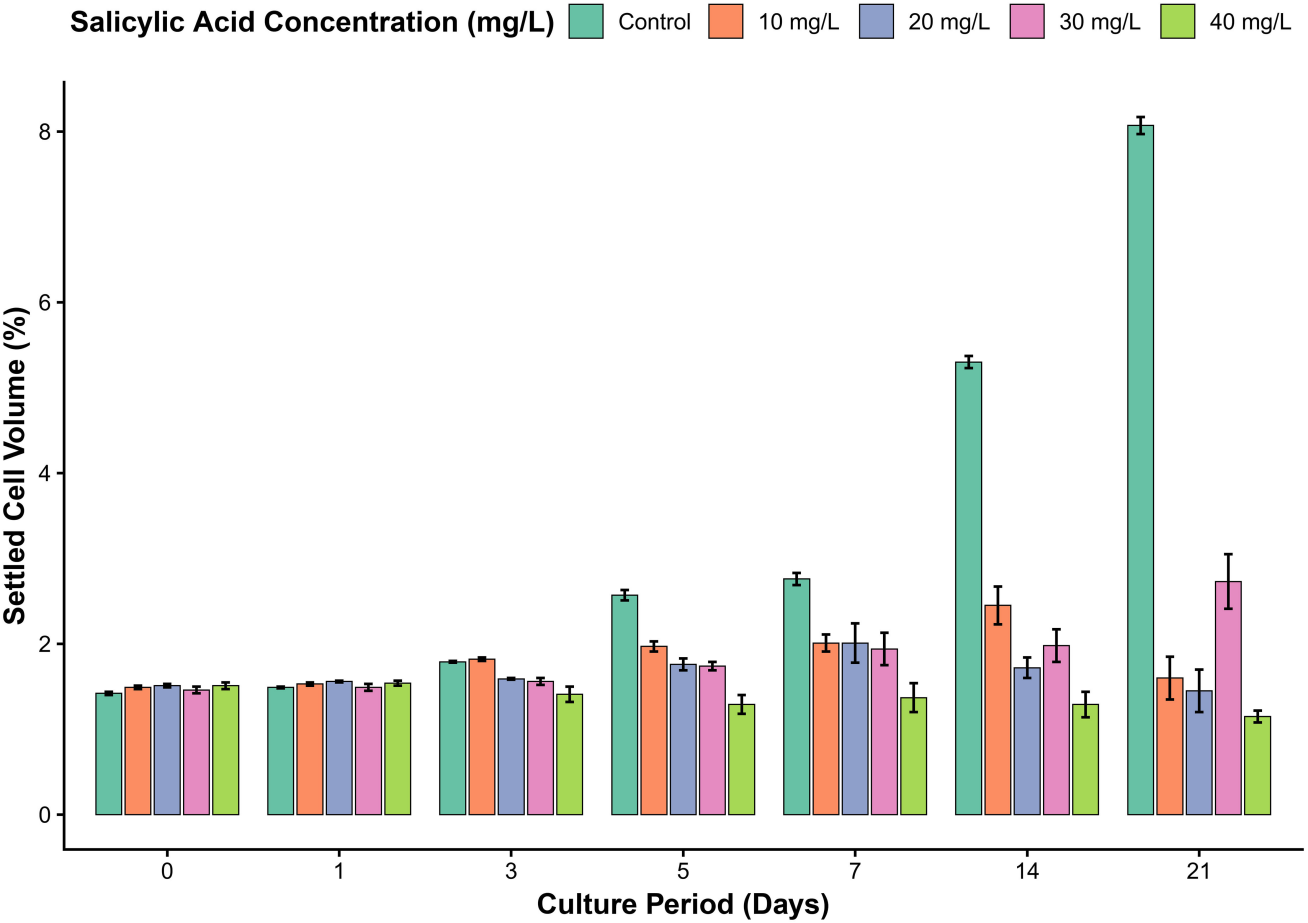
Effect of varying SA concentrations on the biomass production (SCV) in *C. phlomidis* cell suspension cultures developed in liquid B5 medium with 0.5 mg/L IAA, 1.0 mg/L BAP and 1.5 mg/L 2,4-D.

**Figure 7:**
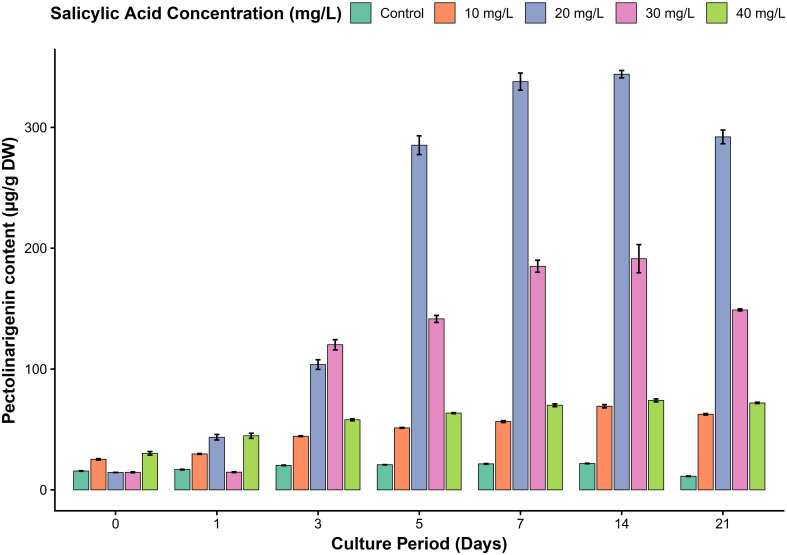
Effect of varying SA concentrations on the Pectolinarigenin (PEC) yield in *C. phlomidis* cell suspension cultures developed in liquid B5 medium with 0.5 mg/L IAA, 1.0 mg/L BAP and 1.5 mg/L 2,4-D.

**Figure 8:**
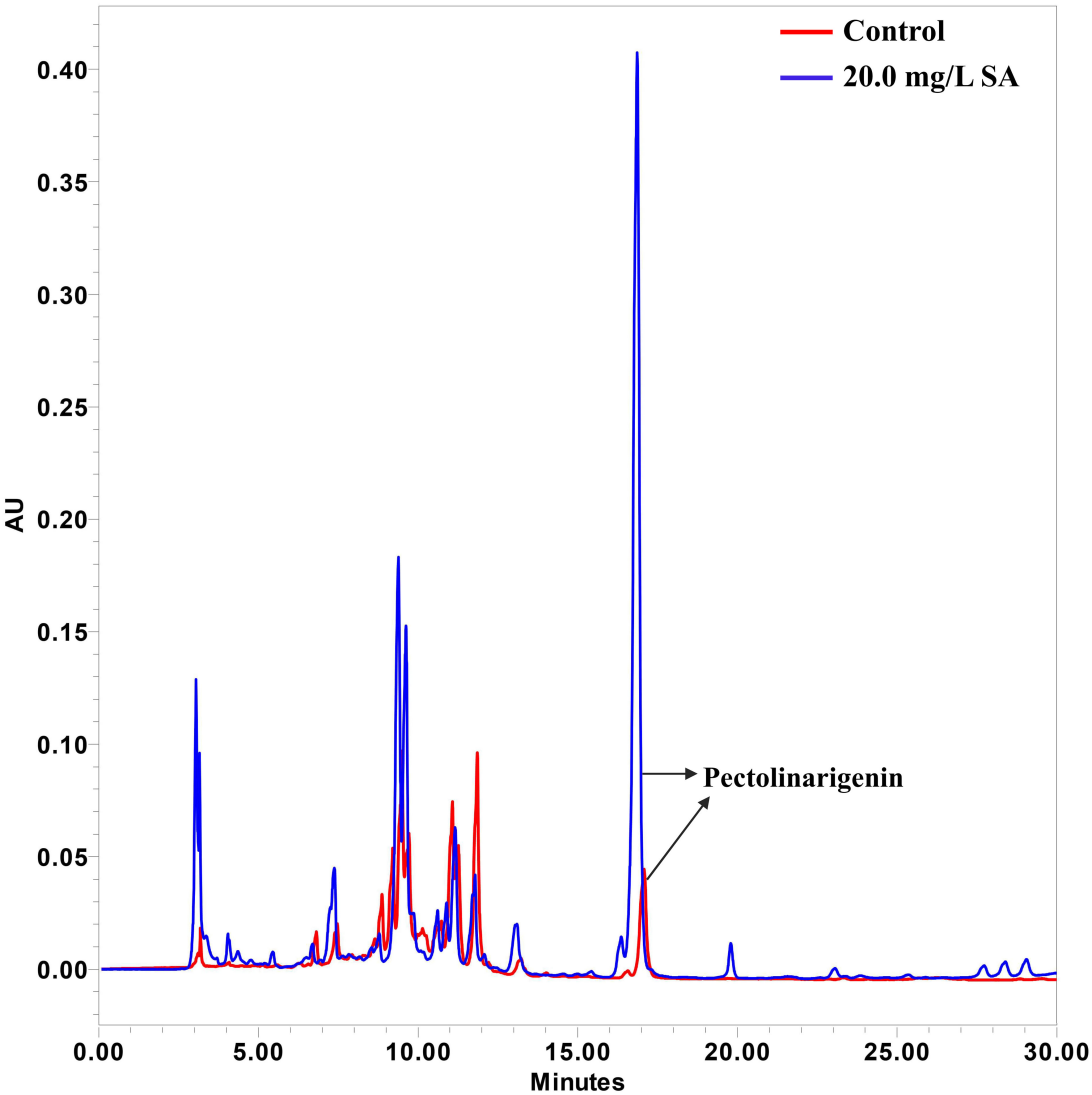
UPLC overlay chromatograms of PEC in control cultures and cultures treated with 20.0 mg/L salicylic acid on 14^th^ day.

The results demonstrate a distinct divergence between biomass proliferation and secondary metabolite accumulation in response to varying concentrations of SA over a 21-day culture period. Biomass accumulation measured through SCV showed a significant boost from moderate SA elicitation because the 20.0 mg/L SA treatment produced the highest growth peak of approximately 8%, which occurred on the 14^th^ day, while 40.0 mg/L SA groups showed minimal growth, which reached under 2% (**Figure 6**). The PEC content exhibited a strong temporal increase, which occurred in opposite relation to SA concentration and biomass growth (**Figure 7**; **Supplementary Table S1**). The observed reduction in biomass accumulation at higher SA concentrations aligns with findings by Bulgakov et al. (2002), suggesting that elevated salicylic acid levels induce significant physiological stress in *in vitro* cultures. The cultures treated with 20.0 mg/L SA produced their highest metabolite accumulation, which achieved a maximum of approximately 343.94 ± 3.09 µg/g DW at the 14th day and the 7th day (337.84 ± 7.11 µg/g DW) (**Figure 8**; **Supplementary Figures S1, S2**). This is higher than the field-grown plant parts showing PEC quantity of 232.84 ± 8.81 µg/g DW and 20.76 ± 0.90 µg/g DW in leaves and flowers, respectively.

**Figure 9:**
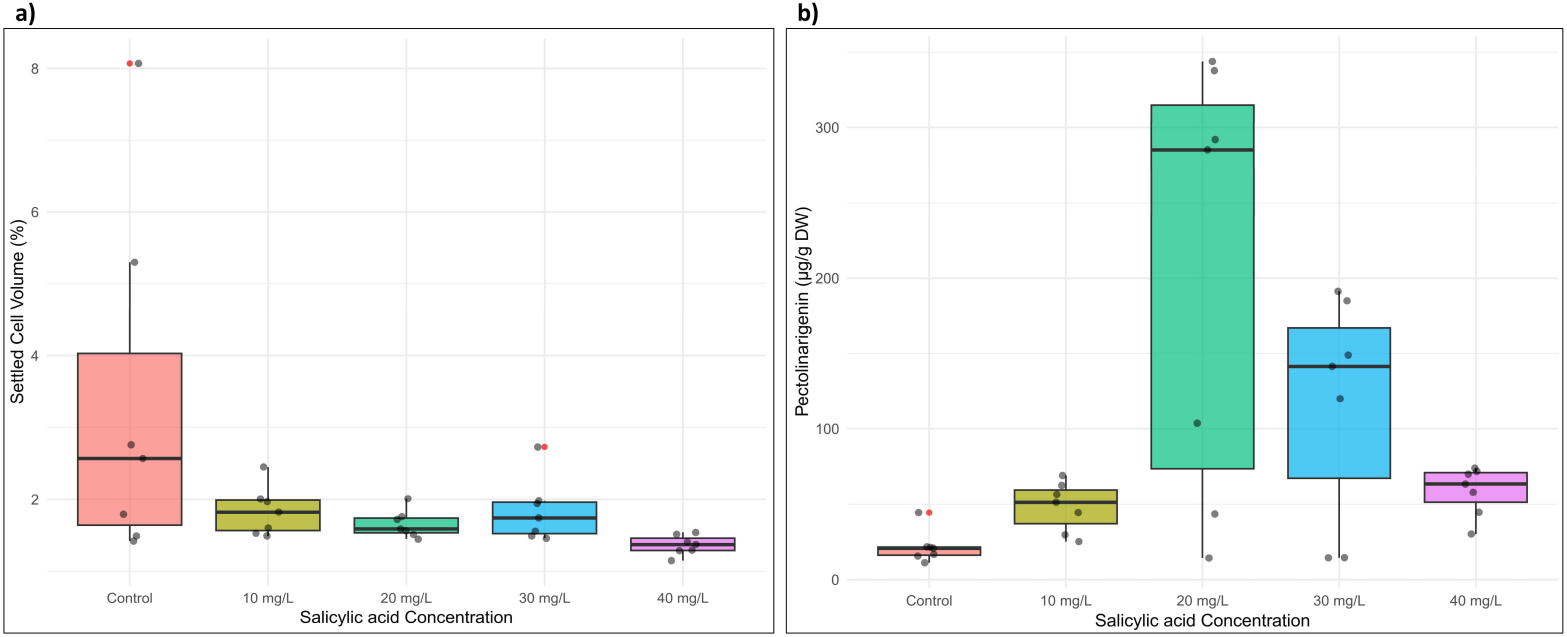
Boxplot analysis of **(a)** biomass growth (SCV %) and **(b)** pectolinarigenin yield in response to different salicylic acid (SA) concentrations.

The boxplot analysis of SCV shows that biomass accumulation was significantly affected by SA concentrations. The control group showed the highest median growth together with the widest range of SCV values, which proved that biomass growth continued at a constant rate without any stress effects. The SCV showed a continuous decrease, which started with the first SA concentration and reached its peak at 40.0 mg/L as the level caused the most significant growth restriction. The study found that SA operates as an effective signal molecule that stimulates secondary metabolism, but when present at high concentrations, it causes a physiological trade-off as it redirects energy from primary growth processes to defense-related biosynthetic pathways (**Figure 9**).

The PEC content showed an increasing response to higher elicitor concentrations until it reached its maximum at 20.0 mg/L. The boxplot shows a major increase in median value together with a broad interquartile range, which shows the metabolite accumulated throughout the entire culture duration. The 30.0 mg/L and 40.0 mg/L treatments produced higher elicitation levels than the control treatment, but the PEC yield decreased at these two higher dose levels compared to the 20.0 mg/L dose as it reached its highest response point.

The concentration-dependent elicitation profile of SA on both biomass accumulation (SCV) and PEC biosynthesis in *C. phlomidis* underscores a highly coordinated, threshold-specific physiological response. Mechanistically, the significant upregulation of PEC content at the optimal concentration of 20.0 mg/L can be attributed to the role of SA as a potent endogenous signaling molecule that triggers the plant’s defense architecture. Exogenous application of SA mimics pathogen attack or environmental stress, initiating a signal transduction cascade that induces the transcription of primary genes governing the phenylpropanoid and downstream flavonoid biosynthetic pathways (Naz et al., 2024; Sun et al., 2022; Roychowdhury et al., 2024). Specifically, SA acts as a transcriptional activator for key rate-limiting enzymes, including Phenylalanine ammonia-lyase (PAL), Chalcone synthase (CHS), and Flavonoid synthase (FLS) (Salinas et al., 2025; Fang et al., 2025; La et al., 2023; Bk, 2026). PAL catalyses the initial deamination of L-phenylalanine to *trans*-cinnamic acid, providing the primary carbon skeleton for all downstream polyphenols (Wu et al., 2025; Zhu et al., 2024). Simultaneously, the upregulation of CHS drives the condensation of p-coumaroyl-CoA with malonyl-CoA to yield chalcones, the core precursors for flavones like pectolinarigenin (Yan et al., 2026; Tariq et al., 2023). The sharp increase in PEC accumulation at 20.0 mg/L suggests that this specific molarity establishes an optimal intracellular signaling threshold, maximizing metabolic flux through these enzymatic checkpoints without crossing into systemic metabolic disruption.

Beyond resource trade-offs, excessive SA accumulation (30.0–40.0 mg/L) induces severe localized cytotoxicity. High intracellular concentrations of SA disrupt the mitochondrial electron transport chain, triggering an uncoupled burst of reactive oxygen species (ROS) that exceeds the buffering capacity of cellular antioxidant enzymes (such as superoxide dismutase and catalase) (Ilyich et al., 2024; Dhiman et al., 2022; Poór et al., 2019). This resulting state of oxidative stress leads to lipid peroxidation of the plasma membrane, loss of mitochondrial membrane potential, and subsequent programmed cell death (PCD) or necrosis within the suspension cultures (Doyle et al., 2010; McCabe and Leaver, 2000). Consequently, the drastic reduction in PEC yield at higher concentrations is not due to a down-regulation of synthesis alone, but rather a direct consequence of compromised cell viability and structural degradation of the biosynthetic machinery within the biomass.

### 3.5 Machine learning models

**Figure 10:**
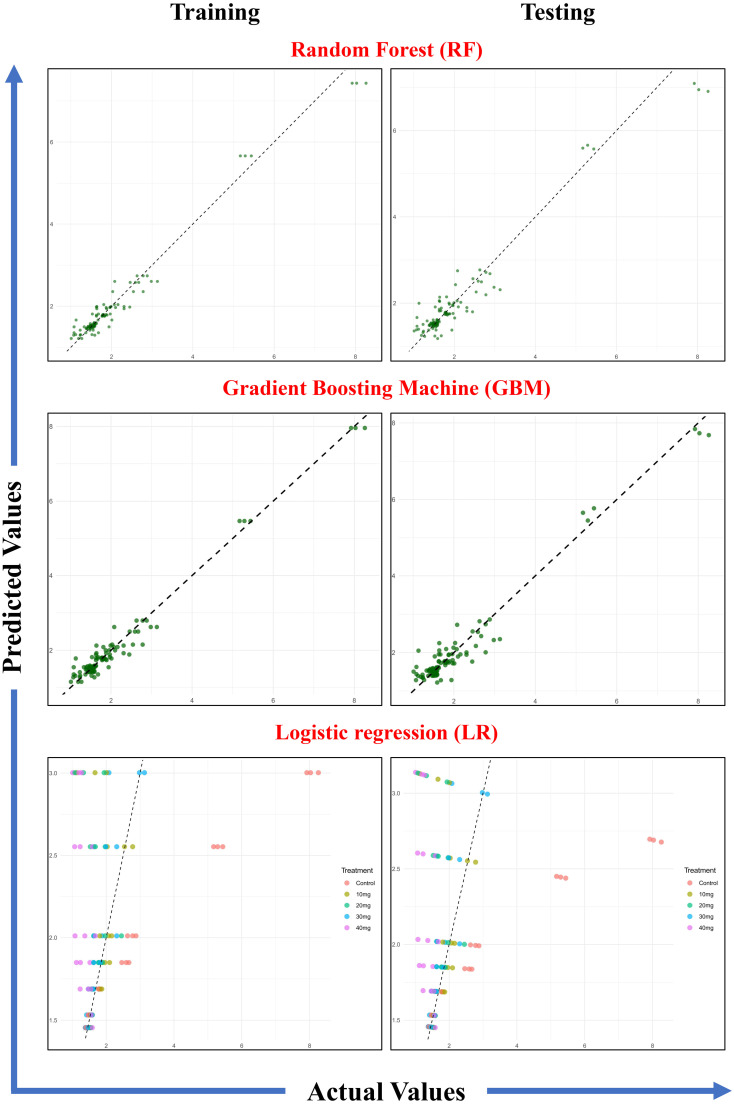
Validation of ML model predictions against observed data for settled cell volume (SCV) in cell suspension cultures under varying salicylic acid concentrations.

**Figure 11:**
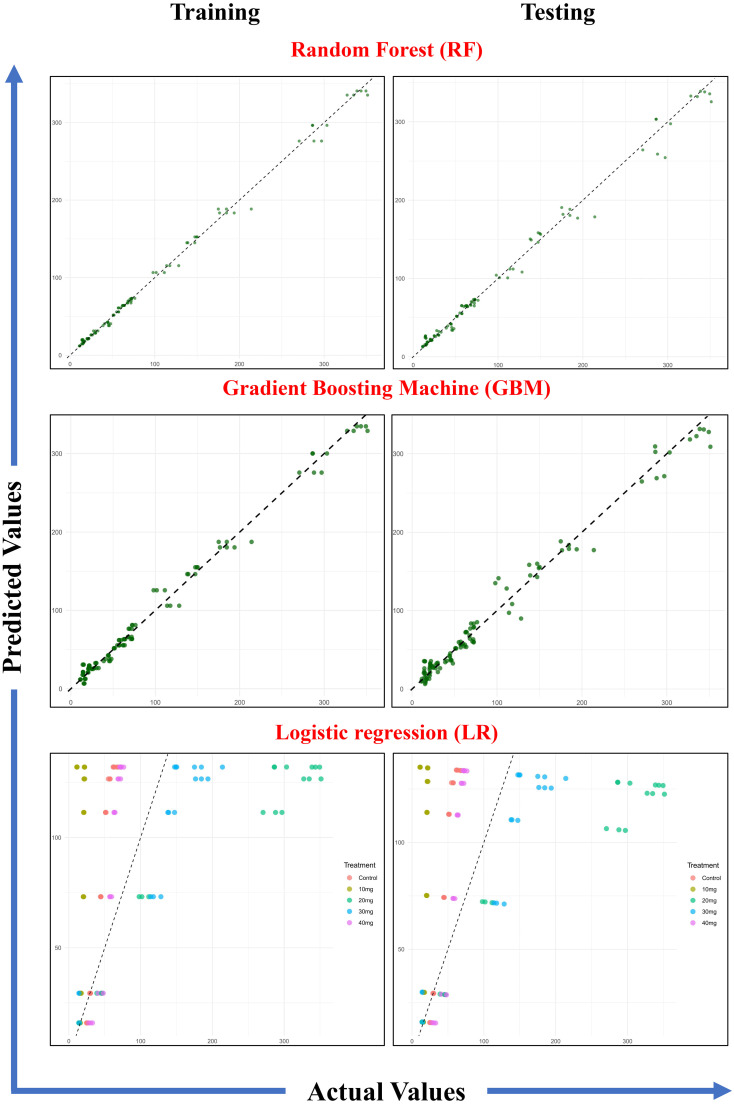
Validation of ML model predictions against observed data for pectolinarigenin (PEC) content in cell suspension cultures under varying salicylic acid concentrations.

To evaluate the predictive capacity of the ML algorithms, the regression performance of Random Forest (RF), Gradient Boosting Machine (GBM), and Logistic Regression (LR) was evaluated across both training and testing datasets (**Table 1**). For the prediction of SCV (%), the tree-based ensemble models demonstrated exceptional robustness and generalization capability. GBM marginally outperformed RF on the testing subset with an R^2^ of 0.953 compared to the R^2^ of 0.942 achieved by RF (**Figures 10**, **11**). Similarly, high-dimensional forecasting of PEC content (µg/g DW) was highly optimized using tree ensembles, where RF exhibited superior precision, yielding an exceptional testing R^2^ of 0.991 with minimized error metrics, closely followed by GBM (R^2^ = 0.981). The negligible performance drop from training to testing phases in both RF and GBM models across both biological targets confirms successful generalization and the absence of overfitting. Conversely, the linear baseline model, Logistic Regression, failed entirely to capture the underlying data architecture, producing critically low testing R^2^ metrics of 0.090 for SCV and 0.194 for PEC content, coupled with severely elevated error values. These findings demonstrate that the experimental parameters influence biomass accumulation and secondary metabolite elicitation through highly complex, non-linear interactions that are effectively modeled by ensemble learning architectures rather than conventional linear approaches.

**Table 1:** ML Algorithms prediction of *in vitro* elicitation of Pectolinarigenin in *C. phlomidis* based on settled cell volume (%) and PEC yield (µg/g DW).

Parameters	Model	Training			Testing		
		R^2^	RMSE	MAE	R^2^	RMSE	MAE
Settled Cell Volume (%)	Random Forest (RF)	0.971	0.223	0.147	0.942	0.323	0.207
	Gradient Boosting Machine (GBM)	0.974	0.203	0.139	0.953	0.274	0.186
	Logistic Regression (LR)	0.171	1.156	0.594	0.090	1.221	0.621
Pectolinarigenin Content (µg/g DW)	Random Forest (RF)	0.996	5.512	3.378	0.991	9.135	5.460
	Gradient Boosting Machine (GBM)	0.991	8.877	6.641	0.981	12.687	9.123
	Logistic Regression (LR)	0.230	82.466	59.765	0.194	84.498	61.312

Machine learning criteria (R^2^, R squared; RMSE, Root mean squared error; MAE, mean absolute error).

**Figure 12:**
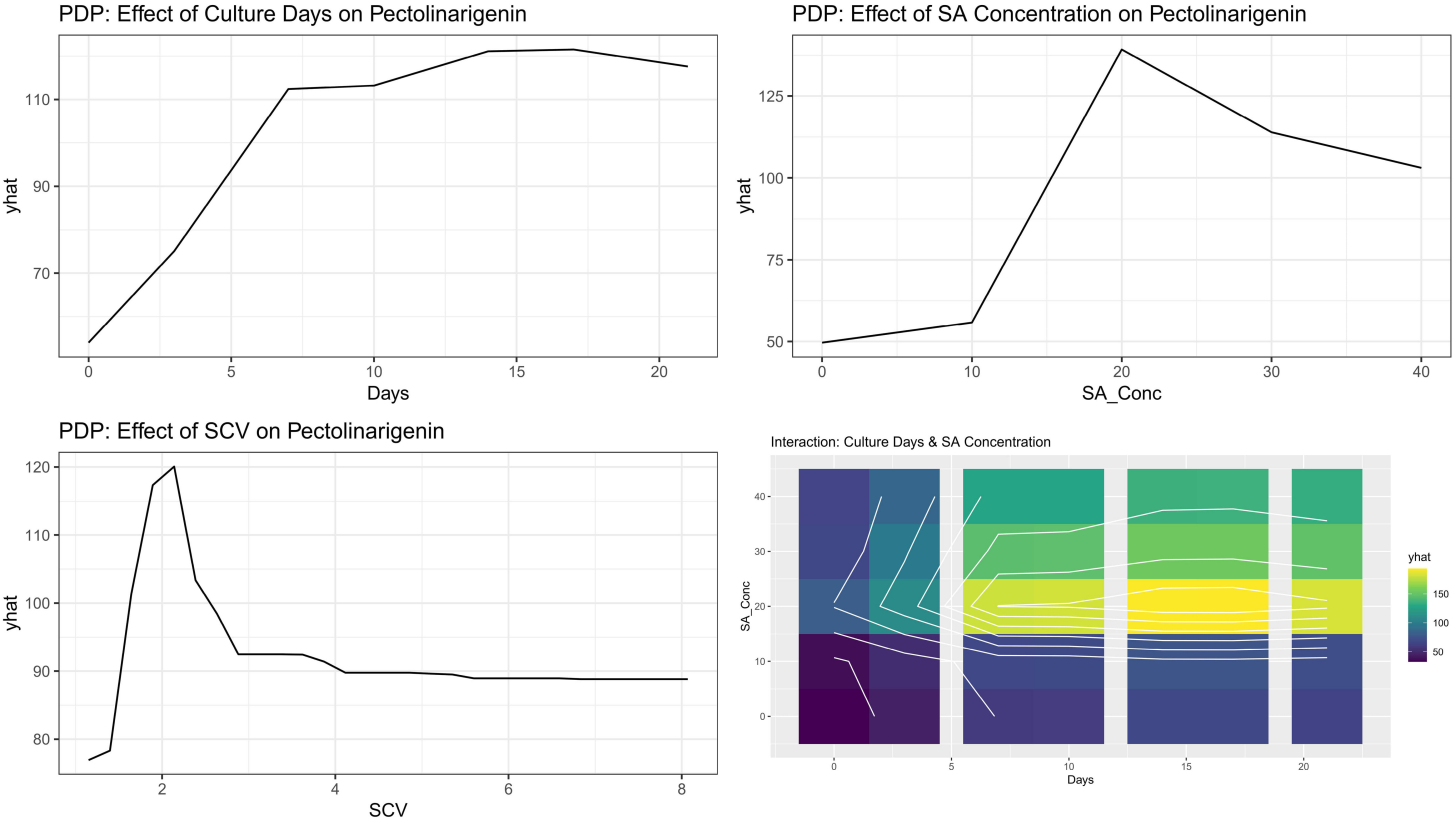
Partial dependence plots (PDP) and interaction analysis of key variables influencing pectolinarigenin production.

The partial dependence analysis results show that Pectolinarigenin accumulation depends on three factors which include culture duration, salicylic acid concentration, and SCV. The temporal profile showed that metabolite yield increased with time during their initial induction period until they reached a maximum between days 14 and 21 which led to a plateau phase that showed a specific time period for optimal biomass production (**Figure 12**). The SA elicitation process showed a unimodal distribution pattern which reached its peak PEC productivity at the 20.0 mg/L concentration level. The presence of higher concentrations above this point resulted in decreasing effects which occurred due to concentration-dependent cytotoxicity and feedback inhibition that affected biosynthetic pathways. The SCV response showed high sensitivity because it reacted strongly to a specific narrow peak that occurred at 2.0 whereas SCV percentages decreased when values surpassed 4.0. The interaction modeling results showed that the “hot zone” synergy created an area which produced the highest predicted pectolinarigenin yields through the combination of 14-day medium-term incubation and 20.0 mg/L SA supplementation that formed specific conditions for maximum secondary metabolite production.

## 4 Limitations and future perspectives

The present study successfully established a standardized protocol for *C. phlomidis* cell suspension cultures using Gamborg B5 basal medium supplemented with specific PGRs (IAA, BAP, and 2,4-D). There are certain limitations remain to be addressed in future research. Primarily, while the currently used PGRs combinations provided a robust baseline, further fine-tuning of the microenvironmental conditions is essential to maximize macro-scale biomass accumulation and accelerate target secondary metabolite enrichment. Furthermore, while the tree-based ML ensembles demonstrated exceptional predictive accuracy in forecasting culture dynamics, the integration of optimization algorithms such as Genetic Algorithms (GA) or Response Surface Methodology (RSM) with the best performing models requires systematic biological validation to confirm empirical efficacy. By integrating these advanced computational frameworks with empirical validation, future studies can dynamically map and navigate the non-linear interaction spaces of salicylic acid elicitation. This will allow for the algorithmic pinpointing of absolute optimal elicitor treating strategies, ultimately maximizing both biomass and pectolinarigenin productivity for sustainable scale-up in bioreactor systems.

## 5 Conclusion

A comprehensive study of salicylic acid on the biomass and pectolinarigenin yield has been performed in *C. phlomidis* cell suspension cultures. The standardised cell suspension cultures were developed following the pectolinarigenin production observed in specific SA concentrations. These findings establish that while SA can be used to manipulate culture dynamics, a strategic balance must be attained between biomass expansion and the induction of defense-related compounds to optimize total volumetric productivity in *C. phlomidis* cultures. The Machine learning evaluation identified Random Forest and Gradient Boosting Machine as the most suitable model for predicting and characterizing the effects of salicylic acid elicitation in these cell suspension cultures. By integrating predictive machine learning architectures with biotechnological frameworks, this study directly aligns with UN Sustainable Development Goal 9 (Industry, Innovation, and Infrastructure) by fostering innovative, data-driven methodologies for scalable and optimized bioproduction.

## Data Availability

The raw data supporting the conclusions of this article will be made available by the authors, without undue reservation.
