# Supplementary material for: Enhancing the pectolinarigenin production in Clerodendrum phlomidis L. f. cell suspension cultures and machine learning-based predictive modeling
Source: Front Plant Sci. 2026 Jun 18;17:1866545. doi: 10.3389/fpls.2026.1866545 (PMC13323313; doi:10.3389/fpls.2026.1866545)
Supplement: Supplementary file 1 [file Table1.docx]

| **Day** | **SA Concentration (mg/L)** | **Settled Cell Volume (SCV (%))** | **PEC Content (µg/g DW)** |
| --- | --- | --- | --- |
| 0 | Control | 1.42±0.02hij | 15.67±0.24lm |
|  | 10.0 | 1.49±0.02hij | 25.33±0.55mn |
|  | 20.0 | 1.51±0.02ghij | 14.41±0.08mn |
|  | 30.0 | 1.46±0.04hij | 14.53±0.4mn |
|  | 40.0 | 1.51±0.04ghij | 30.28±1.51l |
| 1 | Control | 1.49±0.01hij | 16.84±0.31l |
|  | 10.0 | 1.53±0.02ghij | 29.74±0.4mn |
|  | 20.0 | 1.56±0.01efghij | 43.59±2.32k |
|  | 30.0 | 1.49±0.04hij | 14.68±0.36mn |
|  | 40.0 | 1.54±0.03fghij | 44.73±2.1k |
| 3 | Control | 1.79±0.01defgh | 20.33±0.22k |
|  | 10.0 | 1.82±0.02defgh | 44.45±0.25lmn |
|  | 20.0 | 1.59±0.01defghij | 103.69±3.97f |
|  | 30.0 | 1.56±0.04efghij | 120.05±4.26e |
|  | 40.0 | 1.41±0.09hij | 57.98±0.85ij |
| 5 | Control | 2.57±0.06c | 20.82±0.15jk |
|  | 10.0 | 1.97±0.06def | 51.33±0.29lmn |
|  | 20.0 | 1.76±0.07defgh | 285.19±7.74b |
|  | 30.0 | 1.74±0.05defgh | 1414.44±2.94d |
|  | 40.0 | 1.29±0.11ij | 63.52±0.45hi |
| 7 | Control | 2.76±0.07c | 21.5±0.33ij |
|  | 10.0 | 2.01±0.1d | 56.55±0.79lmn |
|  | 20.0 | 2.01±0.23d | 337.84±7.11a |
|  | 30.0 | 1.94±0.19defg | 185.03±4.99c |
|  | 40.0 | 1.37±0.17hij | 70.02±1.11gh |
| 14 | Control | 5.3±0.07b | 21.78±0.19gh |
|  | 10.0 | 2.45±0.22c | 69.2±1.38lmn |
|  | 20.0 | 1.72±0.12defghi | 343.94±3.09a |
|  | 30.0 | 1.98±0.19de | 191.26±11.71c |
|  | 40.0 | 1.29±0.15ij | 73.99±1.25g |
| 21 | Control | 8.07±0.1a | 11.28±0.15hi |
|  | 10.0 | 1.6±0.25defghi | 62.56±0.64n |
|  | 20.0 | 1.45±0.25hij | 292.02±5.66b |
|  | 30.0 | 2.73±0.32c | 148.93±0.85d |
|  | 40.0 | 1.15±0.07j | 71.98±0.53gh |

**Supplementary Table S1 –** Effect of salicylic acid on settled cell volume (SCV, %) and Pectolinarigenin (PEC) content (µg/g DW) in *C. phlomidis* cell suspension cultures

Values indicate mean ± standard error.


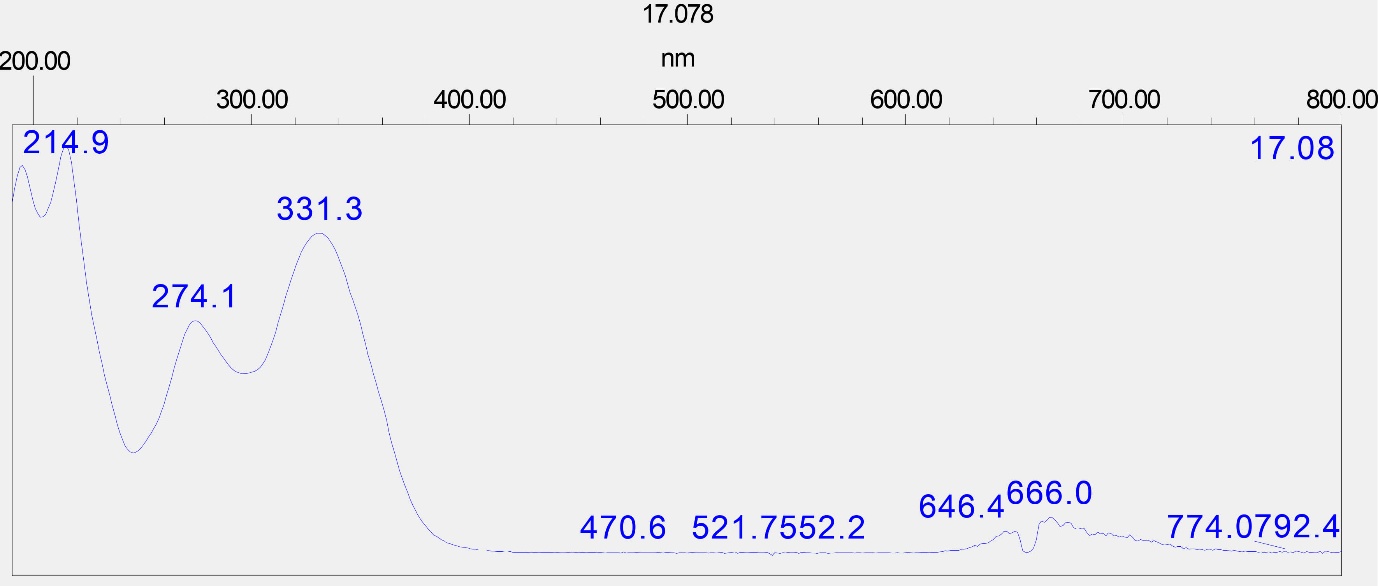


**Supplementary Figure S1** - UV spectrum of control cultures on day 14


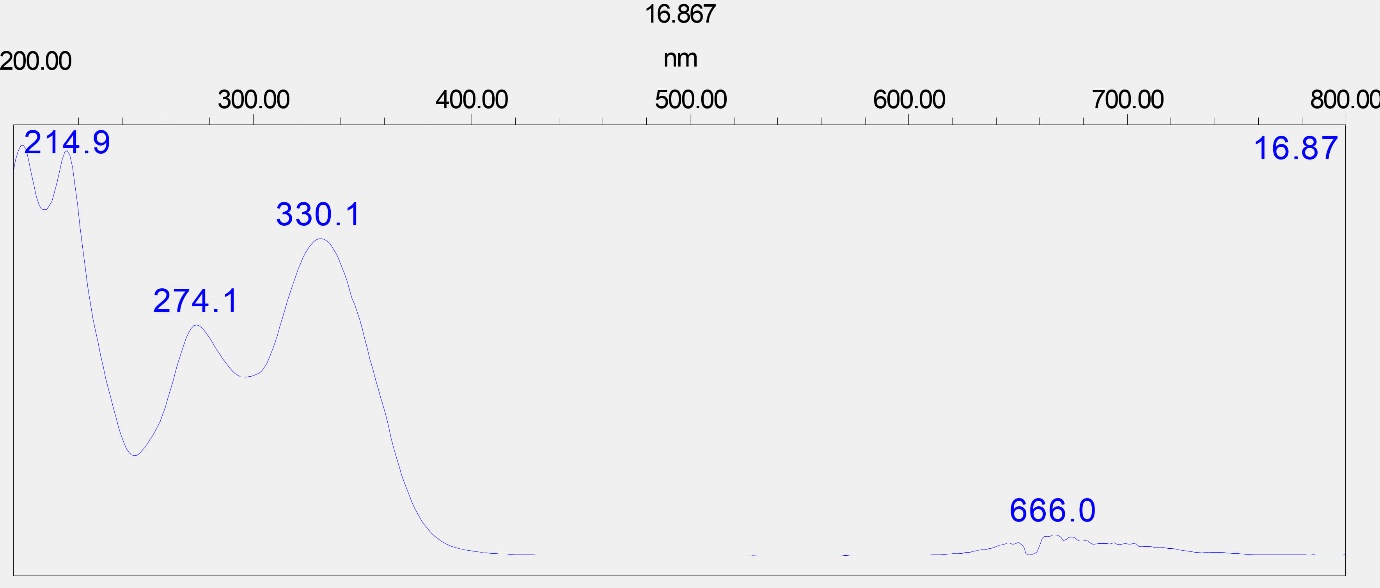


**Supplementary Figure S2** - UV spectrum of 20mg/L SA treated cultures on day 14
